# COVID-19, leprosy, and neutrophils

**DOI:** 10.1371/journal.pntd.0009019

**Published:** 2021-01-07

**Authors:** Veronica Schmitz, Jéssica Brandão dos Santos

**Affiliations:** Laboratório de Hanseníase, Instituto Oswaldo Cruz, Fundação Oswaldo Cruz (FIOCRUZ), Rio de Janeiro, Brazil; Colorado State University - Global Campus, UNITED STATES

## Abstract

Coronavirus Disease 2019 (COVID-19), a disease caused by the betacoronavirus Severe Acute Respiratory Syndrome Coronavirus 2 (SARS-CoV-2), has only recently emerged, while *Mycobacterium leprae*, the etiological agent of leprosy, has endured for more than 2,000 years. As soon as the initial reports of COVID-19 became public, several entities, including the Brazilian Leprosy Society, warned about the possible impact of COVID-19 on leprosy patients. It has been verified that COVID-19 carriers can be either asymptomatic or present varying degrees of severe respiratory failure in association with cytokine storm and death, among other diseases. Severe COVID-19 patients show increased numbers of neutrophils and serum neutrophil extracellular trap (NET) markers, in addition to alterations in the neutrophil-to-lymphocyte ratio (NLR). The absence of antiviral drugs and the speed of COVID-19 transmission have had a major impact on public health systems worldwide, leading to the almost total collapse of many national and local healthcare services. Leprosy, an infectious neurological and dermatological illness, is widely considered to be the most frequent cause of physical disabilities globally. The chronic clinical course of the disease may be interrupted by acute inflammatory episodes, named leprosy reactions. These serious immunological complications, characterized by cytokine storms, are responsible for amplifying peripheral nerve damage. From 30% to 40% of all multibacillary leprosy (MB) patients experience erythema nodosum leprosum (ENL), a neutrophilic immune-mediated condition. ENL patients often present these same COVID-19-like symptoms, including high levels of serum NET markers, altered NLR, and neutrophilia. Moreover, the consequences of a *M*. *leprae*–SARS-CoV-2 coinfection have yet to be fully investigated. The goal of the present viewpoint is to describe some of the similarities that may be found between COVID-19 and leprosy disease in the context of neutrophilic biology.

## Viewpoint

On December 31, 2019, China officially reported the epidemic of a new coronavirus to the World Health Organization (WHO). Due to its high sequence homology to Severe Acute Respiratory Syndrome Coronavirus 1 (SARS-CoV-1), the new coronavirus was named Severe Acute Respiratory Syndrome Coronavirus 2 (SARS-CoV-2). On March 11, 2020, WHO declared that the Coronavirus Disease 2019 (COVID-19), a disease caused by SARS-CoV-2, had become a global pandemic [1].

Leprosy, an ancient infectious disease mainly caused by *Mycobacterium leprae*, was prevalent in Europe until the 16th century and is still endemic in many countries, with over 200,000 new cases reported annually [2]. Leprosy mainly affects the peripheral nervous system, skin, and certain other tissues. The clinical presentation varies from few to widespread lesions. The leprosy poles have clinical, microbiological, and immunological linkage. Clinically, patients may be characterized as paucibacillary when they have less than 5 skin lesions with rare bacilli; in contrast, multibacillary leprosy (MB) are characterized by disseminated lesions filled with bacilli.

Leprosy is treated by a combination of the drugs dapsone, rifampicin, and clofazimine comprising multidrug therapy (MDT). To date, there has been no scientific evidence demonstrating the efficacy of any antiviral drugs to treat the pneumonia caused by COVID-19. Furthermore, clofazimine showed an antiviral activity in Vero E6 cells infected with SARS-CoV-2 in vitro [3]. As a result of this, clofazimine in combination with Interferon Beta-1b is now being tested in a clinical trial for the treatment of hospitalized COVID-19 patients (NCT04465695).

At some point, the leprosy chronic course may be interrupted by acute inflammatory episodes referred to as leprosy reactions. These reactions represent a prominent aspect of the disease due to their ability to accelerate and intensify neural damage and dysfunction. The 2 main types of reaction are referred to as type-1 reaction or reversal reaction (RR) and type-2 reaction or erythema nodosum leprosum (ENL), each with its own distinct characteristics. Several risk factors have already been described for leprosy reactions, including coinfections with other bacteria, parasites, or even viruses [4]. Thus, SARS-CoV-2 infection in leprosy patients has raised important questions about incidence and/or severity of these reactions. In addition, the stress caused by the COVID-19 pandemic and the difficulty in accessing anti-reactional treatment could amplify the leprosy physical disabilities and sequelae [5,6].

There are large variety of COVID-19 disease outcomes. Infected individuals present a range of symptoms from their total absence to headaches, fevers, coughs, loss of smell and taste, dyspnea, and death. Although the respiratory form has garnered the most attention due to high mortality rates from compromised lung function, many individuals have also experienced a mainly intestinal form resulting in nausea, cramps, and diarrhea [7].

Severe COVID-19 patients who have pneumonitis in association with acute respiratory distress syndrome show increased pulmonary inflammation, thick airway secretions, elevated levels of pro-inflammatory cytokines (cytokine storm), extensive lung damage, and microthrombosis. This advanced stage of the disease is difficult to control, resulting in a large number of deaths [8–11]. However, it is still unclear what ultimately is responsible for triggering the cytokine storms seen in these patients.

It has been proposed that the severity of the COVID-19 host response may be primarily due to an aberrant activation of neutrophils in the peripheral blood. Blood cells count of COVID-19 patients revealed a gradual increase in the numbers of white blood cells, the neutrophil percentage, absolute neutrophil count, and neutrophil-to-lymphocyte ratio (NLR) according to the worsening of the disease [7,8,12]. In addition, autopsied lung samples of 3 COVID-19 patients showed neutrophilic infiltration in the pulmonary capillaries, acute capillaritis with fibrin deposition, neutrophilic leakage in the alveolar space, and neutrophilic mucositis [13]. The proteomic profiling of blood obtained from hospitalized patients with COVID-19 detected a neutrophilic activation signature capable of identifying critically ill patients and mortality [14].

Zuo and colleagues [15] demonstrated that COVID-19 patients present high serum levels of free DNA, myeloperoxidase-DNA (MPO-DNA) complexes, and citrunylated histone H3 (Cit-H3). The last 2 components are markers of neutrophil extracellular traps (NETs) [15]. The authors also reported that free DNA strongly correlated with acute-phase proteins, including C-reactive protein (CRP), D-dimer, lactate dehydrogenase, and the absolute numerical neutrophilic count. Cit-H3 levels correlated with the platelet levels. Both free DNA and the DNA–histone complex were elevated and associated with hospitalized patients who had received mechanical ventilation versus those hospitalized without this type of intervention. Finally, COVID-19 patient serum induced the release of NETs in neutrophils from healthy donors in vitro. The clinical relevance of all these findings is corroborated by many of the ongoing clinical trials targeting NETs in COVID-19 patients [13].

Didangelos [16] used a gene network approach on recently published data sets to identify possible COVID-19 inflammatory mechanisms and bioactive genes. The first data set was obtained via a single-cell RNA sequence derived from human tissue that identified a neutrophilic-response signature and relevant inflammatory genes. The “neutrophilic degranulation” network was found to be the main expanded ontology. The second data set analysis, carried out in A549 lung alveolar cells infected by SARS-CoV-2, revealed that infected cells expressed neutrophilic-attracting chemokines. The last analysis used the transcriptome of bronchoalveolar lavage fluid cells from 2 hospitalized COVID-19 patients, identifying the up-regulation of neutrophilic-enriched genes and neutrophilic chemokines [16]. Yet, the immunopathological mechanisms driving COVID-19 remain nebulous, and the contribution of recruited neutrophils and related activities also require further study.

ENL is a serious immunological complication affecting between 30% to 40% of all MB patients [17]. Generally speaking, the incidence of ENL is often higher roughly 2 years after completing MDT. Even so, ENL may occur at any time either before, during, or after discharge from treatment. Cytokine storms and high levels of inflammatory serum mediators such as CRP, serum amyloid A, and pentraxin 3 have been described in ENL patients [4,18]. Some patients experience multiple episodes and, as such, require the administration of oral corticosteroids and/or thalidomide [18]. It should be noted that there are currently clinical trials using corticosteroids or thalidomide to treat COVID-19 patients [19].

ENL is considered a neutrophilic, immune-mediated condition, and the presence of neutrophils in skin lesions is a hallmark [18]. The transcriptome of ENL lesions revealed a signature of genes involved in neutrophilic recruitment [20]. Two microarray data sets evaluating host gene expression in leprosy were reanalyzed, and the information was integrated to strengthen evidence of differential expression for several genes. The combined data revealed that the genes involved in “neutrophilic degranulation”, “neutrophilic activation”, “iron ion homeostasis,” and “extracellular matrix organization” were more fully expressed in ENL lesions than in nonreactive MB control ones [21]. As mentioned above, the “neutrophil recruitment” pathway was also increased in lung cells exposed to SARS-CoV-2 in vitro.

Similar to COVID-19, patients with ENL present neutrophilia and high NLR [22]. ENL-circulating neutrophils present higher expression of CD64, a neutrophilic activation marker, in comparison to those found in the phenotypes in other clinical leprosy forms [18]. ENL neutrophils also spontaneously release tumor necrosis factor alpha (TNF-α) ex vivo differently from what is observed among nonreactional multibacillary controls [23]. Moreover, ENL patients exclusively present circulating-and-skin-lesion-neutrophilic-releasing NETs together with higher DNA–histone complex serum levels, a NET-derived component, than nonreactive MB controls [24].

Furthermore, from the clinical and epidemiological points of view, COVID-19 and ENL have marked differences even though their systemic neutrophilic activation and transcriptome data are similar. It remains to be seen if the leprosy/SARS-CoV-2 coinfection itself actually triggers the onset of ENL by enhancing the neurological damage leading to physical disabilities. Until now, there have been no published data describing any changes in the rates of incidence, severity, or recurrence of ENL since the COVID-19 pandemic began. Further evaluations are needed to deeply understand the role of neutrophils in COVID-19 and ENL. In this regard, it is hoped that the rapid generation of knowledge concerning COVID-19 could be beneficially applied to ENL patients by affording them access to promising new or repositioned drugs capable of modulating neutrophilic activity.
